# GPER Agonist G-1 Disrupts Tubulin Dynamics and Potentiates Temozolomide to Impair Glioblastoma Cell Proliferation

**DOI:** 10.3390/cells10123438

**Published:** 2021-12-07

**Authors:** Alex Hirtz, Nolwenn Lebourdais, Fabien Rech, Yann Bailly, Athénaïs Vaginay, Malika Smaïl-Tabbone, Hélène Dubois-Pot-Schneider, Hélène Dumond

**Affiliations:** 1 Université de Lorraine, CNRS, CRAN, F-54000 Nancy, France; alex.hirtz@univ-lorraine.fr (A.H.); nolwenn.lebourdais@univ-lorraine.fr (N.L.); fabien.rech@univ-lorraine.fr (F.R.); yann.bailly@univ-lorraine.fr (Y.B.); athenais.vaginay@loria.fr (A.V.); helene.dubois-pot-schneider@univ-lorraine.fr (H.D.-P.-S.); 2 Université de Lorraine, CHRU-Nancy, Service de Neurochirurgie, F-54000 Nancy, France; 3 Université de Lorraine, CNRS, Inria, LORIA, F-54000 Nancy, France; malika.smail@loria.fr

**Keywords:** glioblastoma, GPER agonist, G-1, microtubule-targeting agent, microtubule dynamics, proliferation, temozolomide

## Abstract

Glioblastoma (GBM) is the most common brain tumor in adults, which is very aggressive, with a very poor prognosis that affects men twice as much as women, suggesting that female hormones (estrogen) play a protective role. With an in silico approach, we highlighted that the expression of the membrane G-protein-coupled estrogen receptor (GPER) had an impact on GBM female patient survival. In this context, we explored for the first time the role of the GPER agonist G-1 on GBM cell proliferation. Our results suggested that G-1 exposure had a cytostatic effect, leading to reversible G2/M arrest, due to tubulin polymerization blockade during mitosis. However, the observed effect was independent of GPER. Interestingly, G-1 potentiated the efficacy of temozolomide, the current standard chemotherapy treatment, since the combination of both treatments led to prolonged mitotic arrest, even in a temozolomide less-sensitive cell line. In conclusion, our results suggested that G-1, in combination with standard chemotherapy, might be a promising way to limit the progression and aggressiveness of GBM.

## 1. Introduction

Gliomas are one of the most common groups of primary brain tumors of the central nervous system (CNS) in adults. Their classification is based on a combination of histological and molecular features for prognostic purposes [1]. The grades range from 1 to 4: grade 4 designating glioblastoma (GBM), IDH-wildtype, which are fast-growing tumors with anaplastic foci [2].

Their incidence, constantly increasing, is estimated at 6/10^5^. The standard treatment for patients with primary GBM is an as wide as possible surgical resection followed by concomitant radiochemotherapy followed by maintenance chemotherapy by temozolomide for 6–12 months and when possible, addition of tumor-treating field during maintenance chemotherapy [3,4,5]. Despite extensive treatment, most tumor recurrences occur within a few months in the excision margins of the irradiated volume, and lead to death within 21 months after diagnosis [4]. The 5-year survival rate is less than 5% [6].

Although little is known about GBM etiology, numerous sources indicate that women are 50% less likely to develop a GBM than men and respond better to standard treatment [7,8]. Thus, the circulating estrogens produced by the gonads or those synthetized locally in the brain could play a protective role against gliomagenesis [9]. This hypothesis is supported by observations linking exogenous hormone intake to a statistical increase in survival [7,10]. In addition, several preclinical studies confirmed this protective role of estradiol in female rodents but not in males, suggesting a differential expression and/or signaling mechanisms through estrogen nuclear receptor (nER), ERα (encoded by the ESR1 gene) or ERβ (encoded by ESR2), or the membrane receptor GPER (G-protein-coupled estrogen receptor 1) between sexes.

GPER expression has been widely detected in normal rat brain [11,12,13,14,15]. In human brain, GPER distribution appeared similar in both sexes but remains to be determined in detail. Several studies indicated that GPER was functional in neurons might oppose the development of neurodegenerative disorder (for review, see [16]). GPER regulated autophagy in astrocytes [17], alleviated inflammation in microglia and stabilized the blood–brain barrier to ensure neuroprotection after ischemia [18,19]. However, no data have been published so far about a potential role for GPER expression and/or activity in brain tumors.

GPER is a 7 transmembrane domain protein, located mainly at the plasma membrane when N-terminal glycosylated and correctly folded and/or in the endoplasmic reticulum membrane [20]. Ligand stimulation of GPER led to a conformational change of the receptor, allowing a direct interaction between GPER and heterotrimeric Gαs proteins and subsequent activation of various non-genomic signaling pathways [21,22,23,24]. Recent immuno-histological studies indicated that the non-glycosylated P16L variant of this receptor could also be found into the nucleus where it bound gene promoters as a nuclear transcription factor-like molecule [25,26].

Benefits against GBM aggressiveness were reported for estrogenic compounds, such as 2-methoxyestradiol, which might be produced locally into the brain and with high affinity for GPER [27,28,29,30,31]. The already well-known nER antagonist tamoxifen that acted as GPER agonist was tested alone or in combination with standard chemotherapy to treat GBM [32,33,34]. Therefore, the GPER-selective agonist G-1 (CAS No. 881639-98-1) or antagonists G15 (CAS No. 1161002-05-6) and G36 (CAS No. 1392487-51-2, which displayed an improved nER counter selectivity) could be regarded as compounds of interest to modulate GBM cell growth and invasiveness [35,36,37]. It is important to note that, while the anti-cancer effects of G-1 have been demonstrated as GPER-dependent in many solid tumors, GPER-selective activation by G-1 was also often hinted but remained to be formally and experimentally substantiated in many cases.

In this study, we first highlighted the beneficial effect of a high GPER expression for overall survival, especially for female patients. We showed that GPER was expressed in male and female GBM cell lines and localized mainly in the cytoplasm and at the plasma membrane. We explored GPER agonist G-1 effects on cell proliferation. G-1 exposure triggered a transient proliferation arrest, due to G2/M blockade through aberrant microtubules dynamics, even independently of GPER. A combination of G-1 and TMZ treatments led to a prolonged mitotic arrest. Our data suggested that G-1 could be a promising therapeutic compound in GBM that potentiated standard treatments to counteract growth and aggressiveness.

## 2. Materials and Methods

### 2.1. Cell Culture and Treatment

LN229 (CRL-2611) and U251 cells lines were obtained from ATCC (American Type Culture Collection, Manassas, VA, USA) and Sigma-Aldrich (Saint-Quentin-Fallavier, France), respectively. LN229 and U251 cells stably expressing eGFP were obtained in the CRAN laboratory following lentiviral infection. All cell lines were routinely cultured in DMEM (Dulbecco’s modified eagle medium), phenol red-free (Gibco) supplemented with 10% decomplemented FBS, 1% essential amino acids (Sigma-Aldrich), 0.5% non-essential amino acids (Sigma-Aldrich), 0.4% vitamins (Sigma-Aldrich), 1.25% sodium pyruvate (Sigma-Aldrich) and 1% streptomycin/penicillin (Sigma-Aldrich) at 37 °C in a humidified 5% CO_2_ atmosphere. For each experiment, the cells were trypsinized, counted with Thoma cell counting chamber, seeded, and grown for 24 h. Then, the cells were deprived from steroid hormones for 24 h in 10% charcoal-stripped FBS medium. Thereafter, the cells were treated as indicated in the figure legends. G-1, G15 and G36 were purchased from Tocris-BioTechne (Noyal Chatillon sur Seiche, France). Temozolomide and colchicine were purchased from Sigma-Aldrich.

### 2.2. MTT(3-(4,5-Dimethylthiazol-2-yl)-2,5-diphenyltetrazolium Bromide) Assay

Briefly, 500 cells were seeded per well and cultured in 96-well plate in the standard growth medium. After 24 h, the cells were cultured in charcoal-stripped-depleted FBS medium and further exposed for 24 h, 48 h, 72 h, 96 h, 144 h or 192 h to DMSO 0.01% as a vehicle control, G-1 or TMZ. Thereafter, 50 µL of MTT solution (2.5 mg/mL in PBS) was added to each well for 3 h in the dark at 37 °C and 5% CO_2_ atmosphere. After 3 h, the medium was replaced by pure DMSO in each well. The absorbance representing cellular metabolic activity was measured at 540 nm with a spectrophotometer (Multiskan Ascent—Thermo electron corporation, Waltham, MA, USA).

### 2.3. Colony Formation Assay

Cells were seeded at a density of 500 cells per well in 6-well plates and grown for 48 h in standard medium. The culture medium was then replaced with charcoal-stripped FBS-containing medium for 24 h. Then, cells were treated with DMSO 0.01% or 1 µM G-1 for 72 h, followed by 72 h without G-1 treatment to assess the impact of G-1 on colony growth. The colonies formed were subjected to a 10 min fixation in 4% paraformaldehyde and a 30 min staining in 0.1% crystal violet. After removing the staining solution, the colonies were air-dried. The clones of at least 300 µm diameter were counted automatically with the ImageJ software (https://imagej.nih.gov/ij/, accessed on 10 May 2021).

### 2.4. Flow Cytometry

Cells grown in charcoal-stripped FBS-containing medium for 24 h were treated with DMSO 0.01% as vehicle control or 1 µM G-1 for 24 h, 48 h, 72 h and 144 h. After centrifugation, the cell pellets were resuspended in phosphate-buffer saline and fixed in 70% cold ethanol overnight at 4 °C. Cells were stained with propidium iodide for 10 min. Cell cycle distribution was analyzed by estimated stained DNA content and analyzed using the Cytoflex (Beckman Coulter, Villepinte, France).

### 2.5. Immunofluorescence

Cells were grown and treated on coverslips, then fixed with a 4% paraformaldehyde Phosphate Buffer Saline solution and incubated with GPER (Santa Cruz, CA, USA; sc-48525), β-tubulin (Cell Signaling, Danvers, MA, USA; 21285S) primary antibodies (1:100 dilution) followed by incubation with Alexa 555 anti-rabbit antibody (Invitrogen, Carlsbad, CA, USA; A21429) (1:1000 dilution) as previously described [38] or with phalloidin-i-Fluor 488 reagent (Abcam, Cambridge, UK; Ab176753). Finally, the cells were incubated with 1 µg/mL Hoechst 33342 (Sigma-Aldrich). Images were obtained with an ImageXpress (Molecular Devices, San Jose, CA, USA) microconfocal imager.

### 2.6. Western Blot Analysis

Western blot analyses were performed as previously described [38]. Briefly, the cells were collected and lysed in RIPA Buffer containing protease and phosphatase inhibitors. After addition of 2× Laemmli (*v*/*v*), the total protein extracts were separated on 12% SDS-PAGE and transferred to 0.45 µm nitrocellulose membranes. Membranes were blocked with 3% BSA in 0.1% Tween 50 mM TBS and then incubated with GPER (Santa Cruz sc-48525) or β-actin (Cell Signaling 4970S) primary antibodies (1:1000 dilution) overnight at 4 °C, followed by incubation with HRP-coupled anti-rabbit (Cell signaling 7074S) secondary antibody (1:10,000). Luminescence was measured with LAS-3000 Imaging System (Fuji). 

### 2.7. Detection of Monomeric and Polymeric Tubulin

Monomeric tubulin was extracted from the cells with microtubule-stabilizing buffer (MSB) containing 0.5% (*v*/*v*) Triton X-100 for 3 min. The monomeric extract was then removed and diluted in 4× Laemmli (*v*/*v*). The remaining cytoskeleton was treated with MSB/Laemmli buffer for 5 min to extract the polymeric tubulin fraction. All extracts were subjected to Western blotting to detect β-tubulin [39].

### 2.8. Time-Lapse Video Microscopy Imaging

Time-lapse video microscopies were performed with ImageXpress (Molecular Devices) microconfocal Imager. LN229 or U251 cells stably expressing eGFP were allowed to attach for 24 h in 24-well glass plates in standard medium, then starved in 10% charcoal-stripped FBS-containing medium for further 24 h. Cells were treated with 1 µM G-1 or DMSO 0.01% as vehicle control followed by time-lapse microscopy imaging every 2 h for a total of 72 h. During the experiments, cells were maintained in a controlled atmosphere for gas, humidity and temperature.

### 2.9. GBM Patient Cohort and Survival Analysis

Gene expression and patient data from GBM patients were downloaded from The Cancer Genome Atlas (TCGA) using TCGAbiolinks (R package) and further processed with R software (open access available online: https://cran.r-project.org/, accessed on 1 November 2021; version 4.0.2). Data from patients presenting primary GBM with wild type IDH and ATRX status and who received both chemotherapy and radiotherapy (*n* = 99) were selected. The normalization was performed using DESeq2 (R package). Boxplots of GPER expression level were generated for female (*n* = 30) and male (*n* = 69). Patients were assigned to a high- or low-GPER expression group using the optimal cut-off value obtained by using the maximally selected rank statistics by the surv_cutpoint function of the survminer R package. Kaplan–Meier survival curves and the log rank test were used to compare the survival curves (R survival package). Overall Survival (OS) was defined as the time between the date of surgery and date of death or the date of the last follow-up.

### 2.10. Statistical Analysis

Results are expressed as means ± SD (standard deviation). Statistical significance was evaluated by Student *t*-test for two-by-two comparison or using one-way analysis of variance (ANOVA) followed by Dunnett’s multiple comparison test. Standard deviations and standard errors were indicated on figures as advocated by Altman and Bland [40]. The number of independent experiments performed was indicated as *n* in each figure legend.

## 3. Results

### 3.1. Clinical Relevance of GPER Expression in GBM Tumors

**Figure 1:**
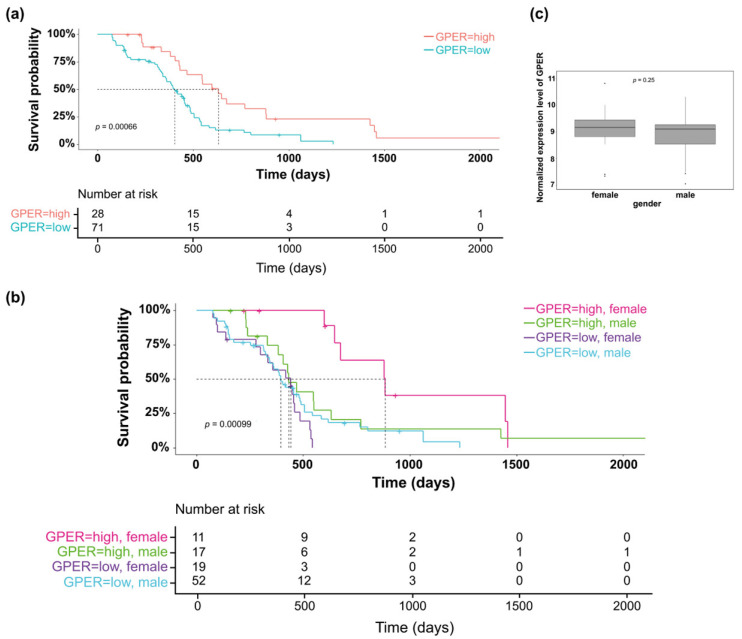
GPER expression in GBM patients. The Kaplan–Meier plots were used to visualize the survival probabilities for the patients diagnosed for a GBM and receiving chemotherapy and radiotherapy (*n* = 99). (**a**) Kaplan–Meier estimate for all patients (male and female) or (**b**) according to patient gender. (**c**) Boxplot showed GPER expression by gender on the 99 patients diagnosed for GBM and receiving chemotherapy and radiotherapy.

Exploration of the GBM project from TCGA database indicated that GPER expression was predictive of overall survival in patients who underwent radiochemotherapy treatment (Figure 1a), but not in the whole patient population (data not shown). Moreover, high GPER expression was significantly of good prognosis for female GBM patients (Figure 1b). Since this finding was not observed for male patients, this could indicate that GPER was differently expressed between both sexes. However, no difference was observed for GPER expression between males and females suggesting that not only the expression level but also the activity of the GPER receptor could be related to survival and better response to anticancer treatment (Figure 1c).

**Figure 2:**
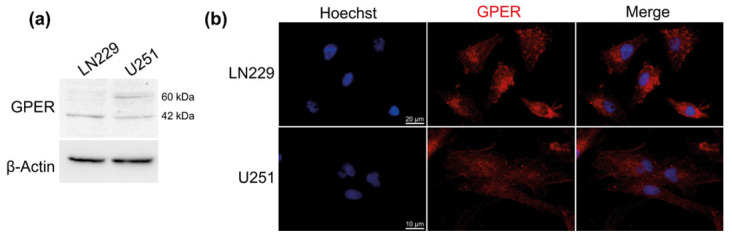
GPER protein expression and localization. (**a**) Representative image of GPER protein expression in LN229 and U251 cells detected by Western blotting. *n* = 4. (**b**) Immunofluorescence staining of LN229 and U251 cells with anti-GPER specific antibody (red). The cell nuclei were stained with Hoechst (blue). Staining was representative of at least three independent experiments.

Therefore, GPER protein expression was assessed by Western blot in LN229 female and U251 male GBM cell lines. Both native (42 kDa) and glycosylated (60 kDa) forms of the GPER protein were detected in GBM cells (Figure 2a). As shown in Figure 2b, GPER localized mainly into the cytoplasm as perinuclear heaps in LN229 cells and was also observed at the plasma membrane in U251 cells.

### 3.2. GPER Agonist G-1 Prevented GBM Cell Proliferation

**Figure 3:**
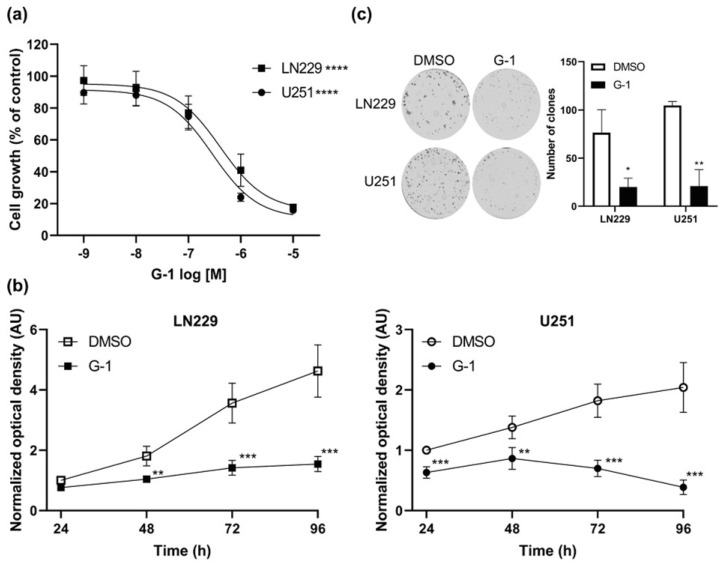
G-1 had cytostatic properties on GBM cells. (**a**) LN229 and U251 cells were treated with indicated concentrations of G-1 for 72 h, and the number of metabolically active cells was determined using MTT assay. *n* = 4. The level of significance was determined using ANOVA with **** indicates *p* < 0.0001. (**b**) LN229 and U251 cells were treated with 1 µM G-1 for 24 h, 48 h, 72 h or 96 h and the number of metabolically active cells was measured using MTT assay. *n* = 4. (**c**) Colony formation assay. Representative images of the number and size of LN229 and U251 clones stained with crystal violet and corresponding quantification of clones of at least 300 µm diameter. *n* = 3. Data are presented as the mean ± SD. The level of significance was determined using Student’s *t*-Test with *** indicates *p* < 0.001, ** *p* < 0.01 and * *p* < 0.05.

Since GPER protein is expressed in LN229 and U251 GBM cells, we evaluated the effect of the GPER specific agonist G-1. A 72 h G-1 exposure prevented cell proliferation in a dose–response manner for both cell lines (Figure 3a). Moreover, treatment with 1 µM G-1 resulted in growth arrest that became significant after 48 h for LN229 and 24 h for U251 cells (Figure 3b). Colony formation assays confirmed that a 72 h exposure to 1 µM G-1 inhibited cell growth compared with DMSO control in both cell lines (Figure 3c).

**Figure 4:**
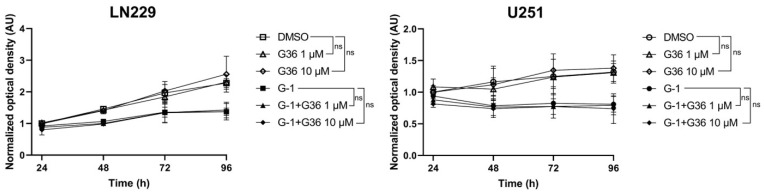
G-1 acted in a GPER-independent manner. LN229 and U251 cells were pretreated for 24 h with 1 µM or 10 µM of the GPER antagonist G36 and then for another 96 h with a combination of 1 µM G-1 and 1 µM G36 or 1 µM G-1 and 10 µM G36. The number of metabolically active cells was determined using MTT assay. *n* = 3. Data are presented as the mean ± SD. The level of significance was determined at 96 h using Student’s *t*-test with ns = non-significant.

In contrast, G15 or G36 antagonists did not trigger any dose–response or time-dependent effect on GBM cell proliferation compared to DMSO-treated ones (Supplementary Figure S1). Moreover, when cells were pretreated for 24 h with the GPER antagonist G36 and then for 96 h with a combination of 1 µM G-1 and 1 µM G36 or 1 µM G-1 and 10 µM G36, the G-1-dependent cytostatic effect was still observed (Figure 4). This strongly suggested that G-1 impact was independent of GPER in GBM cells.

### 3.3. G-1 Exposure Triggered G2/M Cell Cycle Arrest in GBM Cells

**Figure 5:**
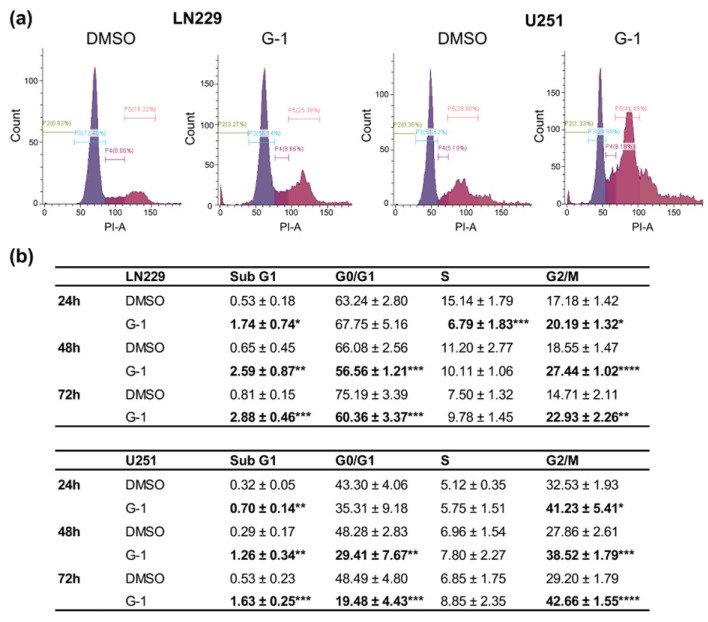
G-1 caused cell cycle arrest in G2/M phase. (**a**) Flow cytometry analyses of the distribution of LN229 or U251 cells in different phases of the cell cycle after 24 h, 48 h and 72 h of treatment with 1 µM of G-1 or DMSO control. Flow cytometry histograms are representative of the 72 h treatment condition. (**b**) Flow cytometry analysis quantifications are presented as the mean ± SD. *n* = 4. The level of significance was determined using Student’s *t*-test with **** indicates *p* < 0.0001, *** *p* < 0.001, ** *p* < 0.01 and * *p* < 0.05.

The effect of G-1 on cell cycle progression was further investigated in LN229 and U251 cells (Figure 5a). Flow cytometry analyses revealed that time-dependent G-1 treatment was able to increase subG1 cell population, which nevertheless remained less than 3% for LN229 and less than 2% for U251 (Figure 5b). G-1 treatment was also able to induce a G2/M arrest at the expense of the G0/G1 in both cell lines after a 24 h treatment. This blockage continued until 72 h of treatment, which seemed to prevent cell division (Figure 5b). The increase in p21 protein expression confirmed the cell cycle arrest (Supplementary Figure S2) even if no variation in cyclin expression was observed, especially cyclin D or cyclin B ones (data not shown).

### 3.4. G-1 Induced a Major Reorganization of Cell Cytoskeleton

**Figure 6:**
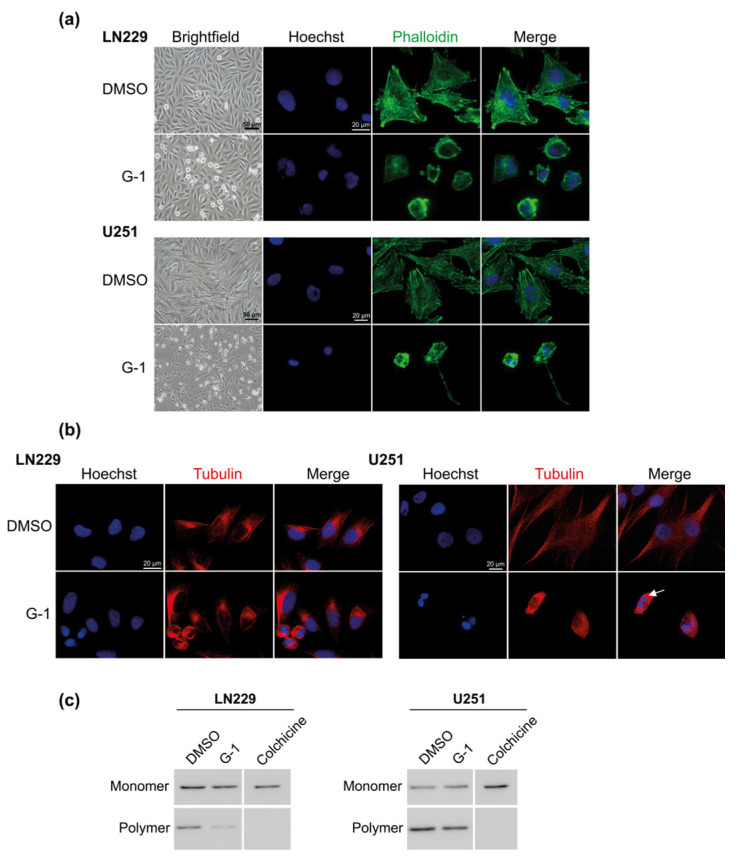
G-1 exposure triggered major changes in cytoskeleton. Representative images of LN229 and U251 cells exposed to DMSO or 1 µM G-1 for 72 h observed in (**a**) brightfield or immunostained with phalloïdin (green) or (**b**) anti-β-tubulin antibody (red). Nuclei were stained with Hoechst (blue). Arrow indicates multiple DNA content. *n* = 4. (**c**) Western blot detection of monomeric and polymeric β-tubulin in cells treated with 1 µM G-1 or DMSO for 72 h. Colchicine (1 µM) was used as positive control for monomeric β-tubulin fraction. *n* = 3.

Concurrently to cell cycle arrest, G-1 triggered a drastic change in cell morphology leading to round but still attached cells (Figure 6a and Supplementary Video S1). Moreover, microtubules immunostained with an anti-β-tubulin antibody appeared shortened and surrounding multiple DNA-rich compartments in round shaped cells after 72 h of treatment, especially in U251 cells (Figure 6b). Measurement of monomeric and polymeric tubulin fractions showed that G-1 treatment increased the abundance of β-tubulin monomers and correspondingly decreased β-tubulin polymers for 29% in LN229 and 32% in U251 cells (*p* < 0.05) (Figure 6c). Consequently, G-1 appeared to impact cell cycle and proliferation through altering cytoskeleton and microtubule dynamics.

**Figure 7:**
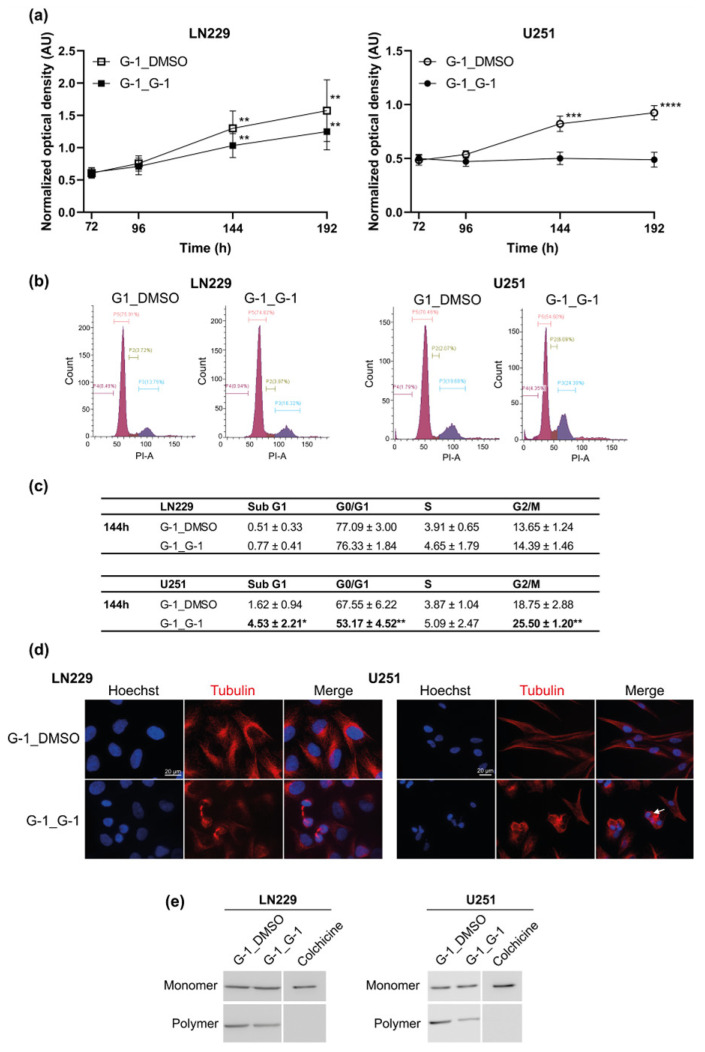
G-1 mediated cytostatic effect was reversible. (**a**) LN229 and U251 cells were treated with 1 µM G-1 for 72 h. G-1 treatment was then replaced by DMSO (G-1_DMSO) as vehicle or renewed (G-1_G-1) for another 120 h and the number of metabolically active cells was determined using MTT assay. *n* = 4. For each treatment, the measured optical density at each time point was normalized and compared to the one of DMSO-treated cells at 72 h for each cell line. (**b**) Flow cytometry analyses of the distribution of LN229 and U251 cells in different phases of the cell cycle after 144 h of 1 µM G-1 or 1 µM of G-1 for 72 h followed by 72 h by DMSO (G-1_DMSO). Flow cytometry histograms are representative of the 144 h treatment condition. (**c**) Flow cytometry analysis quantifications are presented as the mean ± SD. *n* = 5. (**d**) Representative images of LN229 and U251 cells exposed to 1 µM of G-1 for 144 h (G-1_G-1) or 1 µM of G-1 for 72 h followed by 72 h treatment by DMSO (G-1_DMSO) and then stained with an anti-β-tubulin antibody (red). Nuclei were stained with Hoechst (blue). Arrow indicates multiple DNA content. Staining was representative of at least three independent experiments. (**e**) Western blot detection of monomeric and polymeric β-tubulin in cells treated with 1 µM G-1_DMSO or G-1_G-1 for 72 h. Colchicine (1 µM) was used as positive control for monomeric β-tubulin fraction. *n* = 3. The level of significance was determined using Student’s *t*-test with **** indicates *p* < 0.0001, *** *p* < 0.001, ** *p* < 0.01 and * *p* < 0.05.

To check if G-1 exposure could ultimately lead to cell death as a result of altered microtubule dynamics G-1 treatment was renewed (G-1_G-1) or replaced by DMSO (G-1_DMSO) as vehicle for another 120 h. LN229 cells seemed to acquire a G-1 resistance from 144 h of exposure since proliferation resumed, even when G-1 treatment was renewed (Figure 7a–c). MTT assays also indicated that the G-1-dependent cytostatic effect was reversible in both LN229 and U251 cells since G-1_DMSO treated cells resumed proliferation (Figure 7a). This was confirmed by cytometry since the G2/M cell cycle blockade was no longer observed in G-1_DMSO treated cells (Figure 7b,c). During this resumption of proliferation (G-1_DMSO compared to G-1_G-1), cell morphology, nuclear DNA content, cytoskeleton distribution and tubulin polymerization returned to untreated cell phenotype (Figure 7d,e), even if cell extensions appeared stretched in U251 cells.

### 3.5. Additive Effect of G-1 and TMZ Suppressed Cell Proliferation Resumption

**Figure 8:**
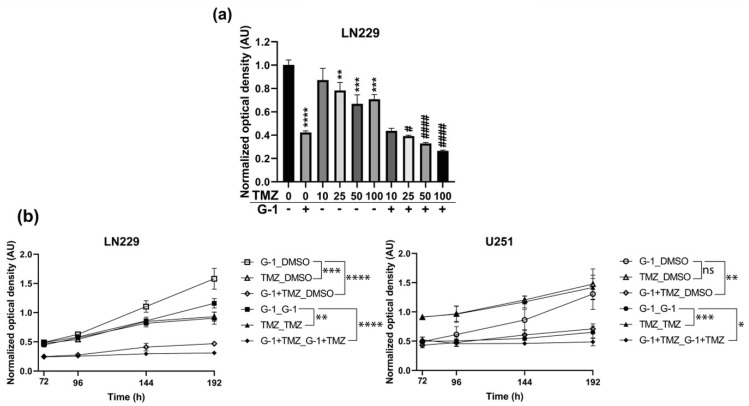
G-1 added to TMZ to prevent cell proliferation. (**a**) LN229 cells were exposed to 1 µM of G-1 for 72 h with or without a dose-dependent TMZ co-treatment and submitted to MTT assay. *n* = 3. The level of significance was determined using ANOVA Dunnett’s multiple comparison test comparing each condition to DMSO (**, ***, ****) or G-1 (#, ####) with ****/#### indicates *p* < 0.0001, *** *p* < 0.001, ** *p* < 0.01, # *p* < 0.05. (**b**) LN229 or U251 cells were exposed to DMSO as vehicle or 1 µM of G-1 for 72 h with or without 100 µM TMZ co-treatment. Initial treatment was either renewed for another 120 h (G-1_G-1; TMZ_TMZ; G-1+TMZ_G-1+TMZ) or replaced by DMSO (G-1_DMSO; TMZ_DMSO; G-1+TMZ_DMSO) and cells were submitted to MTT assay. For each time point, the measured optical density was normalized by the one of DMSO-treated cells at 72 h. *n* = 4. The results are presented as the mean ± SD. The level of significance was determined using Student’s *t*-test with **** indicates *p* < 0.0001, *** *p* < 0.001, ** *p* < 0.01 and * *p* < 0.05, ns = non-significant.

To evaluate the potential benefit of G-1 as an anti-tumor drug in GBM, in combination with the standard TMZ chemotherapy, LN229 and U251 cells were exposed for 72 h to a range of 10 µM to 100 µM TMZ in combination or not with 1 µM G-1. U251 cell growth was not affected by up to 100 µM TMZ (data not shown). LN229 proliferation was reduced by 20–30% by TMZ treatment from 25 µM (Figure 8a). Moreover, the addition of 1 µM G-1 led to an enhanced inhibition of proliferation compared to either G-1 or TMZ alone.

The impact of a 1 µM G-1 and 100 µM TMZ co-treatment on proliferation resumption was also tested on both LN229 and U251 cells. Cells were exposed to either DMSO as vehicle, G-1, TMZ, or the combination of both for 72 h. Then, treatments were renewed or replaced by DMSO for another 120 h. A prolonged exposure to either G-1 or TMZ failed to stop proliferation of LN229 cells, whereas the combination of both G-1 and TMZ triggered a continuous growth inhibition (Figure 7a and Figure 8b). Proliferation resumed similarly after 72 h when G-1 or TMZ treatments were replaced by DMSO (G-1_DMSO or TMZ_DMSO, respectively), whereas a long-term impairment was observed when both G-1 and TMZ were applied during the first 72 h and then replaced by DMSO (G-1+TMZ_DMSO) (Figure 8b). Taken together, those data strongly suggested that TMZ and G-1 could display additive dose- and time-dependent effects on GBM cell proliferation.

U251 cells were not sensitive to a range of TMZ concentrations up to 100 µM, even after 192 h of exposure (TMZ_TMZ, Figure 8b). However, a 192 h G-1 treatment, alone (G-1_G-1) or in combination with TMZ (G-1+TMZ_G-1+TMZ), led to a prolonged proliferation arrest (Figure 7a and Figure 8b). Notably, while cell proliferation resumed when G-1 treatment was replaced by DMSO (G-1_DMSO), long-term proliferation impairment up to 192 h was obtained after a 72 h G-1+TMZ co-treatment, then replaced by DMSO (G-1+TMZ_DMSO, Figure 8b). Therefore, G-1 appeared to potentiate TMZ chemotherapeutic properties to counteract proliferation of TMZ-resistant GBM cells.

## 4. Discussion

G-1 exposure and/or GPER signaling impacted the progression of various hormone-responsive tumors such as ovarian [41,42,43], endometrial [44,45], breast [46] and testicular germ cell tumors. In breast, GPER expression and localization were important factors in tumor progression. In normal breast tissue, triple-negative or high histological grade breast tumors, Samartzis and colleagues [25] detected GPER into the nucleus whereas the level of cytoplasmic (but not nuclear) GPER protein was associated with better overall survival in luminal tumors [47,48]. GPER was known to initiate non-genomic estrogen signaling when localized into the plasma or the endoplasmic reticulum membrane whereas it directly bound gene promoters and modulated transcription into the nucleus [26]. These localization profiles suggested that when a high amount of GPER receptor was able to be activated by estrogens, as in luminal breast tumors, and to trigger non-genomic signaling in the cytoplasm, its activity could contribute to alleviate tumor progression or improve response to anti-cancer treatment.

In the present study, we described for the first time the expression of GPER protein in GBM cell lines and explored its localization. Protein analyses in U251 and LN229 indicated that GPER was expressed in both native (42 kDa) and glycosylated (60 kDa) forms with a greater amount of the latter observed in U251 cells [49,50]. This difference between both cell lines correlated with differential subcellular localization: GPER was mainly cytoplasmic in LN229 but both cytoplasmic and associated with the plasma membrane in U251. Valdivia and coworkers [50] showed that the N-terminal domain residue Asn44 was critical for a mature and functional GPER protein in the plasma membrane. This suggested that the receptor might perform differential functionalities in U251 or in LN229 GBM cells.

In silico analysis of GPER expression across TCGA samples indicated that GBM are one of the tumors that expressed the most GPER mRNA (UALCAN database, [51]). Thus, GPER-dependent signaling could appear favorable since TCGA-GBM data also indicated that a high GPER expression level could be of better prognosis for female (but not male) GBM patients treated with radio-chemotherapy.

U251 and LN229 GBM cells derived from a male or a female tumor, respectively, were exposed to G-1, a synthetic agonist initially selected to specifically trigger GPER activity. G-1 treatment stopped cell proliferation by triggering a G2/M arrest combined to impaired microtubule dynamics in both cell lines, but with slight differences in sensitivity and duration of treatment impact. However, no drastic increase in apoptotic cell number was observed. Cells appeared round shaped and multi-nucleated-like, although not displaying a senescent phenotype (data not shown), which was already observed following 0.25 to 2 µM G-1 in vitro exposure, and often confirmed in vivo by a decrease in xenografted tumor growth, in several solid tumors [41,49,52,53,54]. In breast cancer cells, GPER activation gave conflicting results depending on the relative expression level of nER isoforms, EGFR and GPER itself (see [55] for review). In seminoma and embryonal carcinoma, G-1, 17β-estradiol or xenoestrogen-dependent GPER activity were conversely shown to stimulate cell proliferation in combination with the ERα36 variant [56,57]. GPER even exhibited ligand-independent activity in vitro, depending on its expression level [58]. Therefore, the consequences of the modulation of not only the activity but also the expression of GPER, in combination with other steroid receptor isoforms should be carefully assessed in GBM.

In GBM cells, GPER antagonist G15 or G36 did neither modulate proliferation nor counteract G-1-dependent proliferation arrest, suggesting that observed effects of G-1 on cell phenotype are independent of GPER. The differential response observed between LN229 and U251 cells in terms of intensity and duration of proliferation blockade did not rely on GPER expression, localization or functionality but rather on growth rate, since LN229 doubling time was 1.7-fold higher than U251 in FBS-depleted medium. Therefore, LN229 cells might escape growth arrest earlier than U251. Sixto-Lopez and colleagues [59] also failed to detect any cytotoxicity or proliferative modulation of new GPER antagonists in 2D or 3D GBM stem cell cultures. As assessed by GPER antagonist treatments or GPER targeting siRNA transfection, G-1 effects were also described as GPER-independent in pancreatic ductal adenocarcinoma [52], adrenocortical carcinoma [54] and vascular smooth muscle cells [39], whereas the question is still debated in ovarian [41,49] or breast cancer cells (see [60] for review). A recent study in several eukaryotic systems ex vivo and in vitro in the presence or absence of GPER even stated that neither G-1 nor E2 stimulated its activity [58]. Therefore, G-1, initially designed as a GPER-selective agonist is increasingly described as triggering not only GPER-dependent but also GPER-independent activities.

In most studies, G-1-dependent cancer cell growth inhibition seemed to originate in a failure of tubulin dynamics [39,41,61]. Using a competitive binding assay, Lv and colleagues [61] demonstrated that G-1 bound the colchicine site of tubulin monomers and led to polymerization impairment. In U251 and LN229 cells, we confirmed that G-1 exposure alleviated tubulin polymerization, leading to an increase proportion of monomeric tubulin. Therefore, G-1 might be considered as a new chemotherapeutic drug targeting microtubules.

In a rat model of ischemia/reperfusion, G-1 exposure seemed to improve neuron survival whereas it led to astrocyte apoptosis [16,62]. The toxicity of G-1 toward healthy glial cells surrounding the GBM tumor remains to be tested in vivo.

In ovarian or Leydig cancer cells, G-1 enhanced apoptosis and cell death due to impaired mitotic spindle or multiple spindle aster formation. However, no significant increase in GBM cell death was detected in the present study after G-1 treatment. Unexpectedly, real-time recording of cell proliferation and MTT assays rather indicated that the G-1 blockade of cell proliferation as well as alterations of cell morphology and cytoskeleton were reversible after G-1 treatment was removed. Recent studies highlighted the sensitivity of GBM cells to microtubule-targeting agents and the crucial involvement of cytoskeleton dynamics in mediating TMZ resistance [63,64]. Our data indicated that U251 but not LN229 cells were resistant to moderate dosage of the standard chemotherapeutic agent TMZ. Addition of G-1 to TMZ-treated LN229 cells potentiated TMZ’s effect. Interestingly, the combination of both treatments also led to long term growth arrest duration after compound removal even in the TMZ-resistant U251 cells. Our data supported previous observations suggesting that combination of microtubule-targeting agents, such as G-1 with TMZ, could be a promising therapeutic strategy. Indeed, Xu and coworkers demonstrated the synergic antitumor effect of the combination of Taxol and TMZ on U87 cells [65]. However, Taxol, like many microtubule-targeting drugs, did not cross the blood–brain barrier [66]. Even if the chemical structure of G-1 led us to believe that it could cross the blood–brain barrier, this point needs to be addressed experimentally.

Recently, preclinical studies reported the therapeutic benefit of G-1 treatment on obesity and diabetes in rodents [67]. The therapeutic form of G-1, Tespria™ (Patent Number: US 10,471,047 B2), is currently challenged for treating metabolic disorders in humans. In the future, G-1 should also be re-purposed as an anti-tubulin agent in rodent pre-clinical models of GBM that could act independently of GPER to block cell proliferation. Thereafter, G-1 could be validated as a promising drug to improve GBM treatment efficacy in combination with standard chemotherapy, especially for patients with recurrent GBM and/or TMZ-resistant tumors.

## Data Availability

The results published here are based upon data generated by the TCGA Research Network: https://www.cancer.gov/tcga. Data from TCGA-GBM dataset are available at https://portal.gdc.cancer.gov/projects/TCGA-GBM.

## Supplementary Materials

The following are available online at https://www.mdpi.com/article/10.3390/cells10123438/s1, Figure S1: G15 or G36 GPER antagonists had no effect on GBM cell proliferation. Figure S2: G-1 increased p21 protein expression. Video S1: G-1 altered GBM cell morphology as assessed by time-lapse video microscopy.
