# Supplementary material for: GPER Agonist G-1 Disrupts Tubulin Dynamics and Potentiates Temozolomide to Impair Glioblastoma Cell Proliferation
Source: Cells. 2021 Dec 7;10(12):3438. doi: 10.3390/cells10123438 (PMC8699794; doi:10.3390/cells10123438)
Supplement: Supplementary file 1 [file cells-10-03438-s001.zip › Supplementary Figures.pdf]

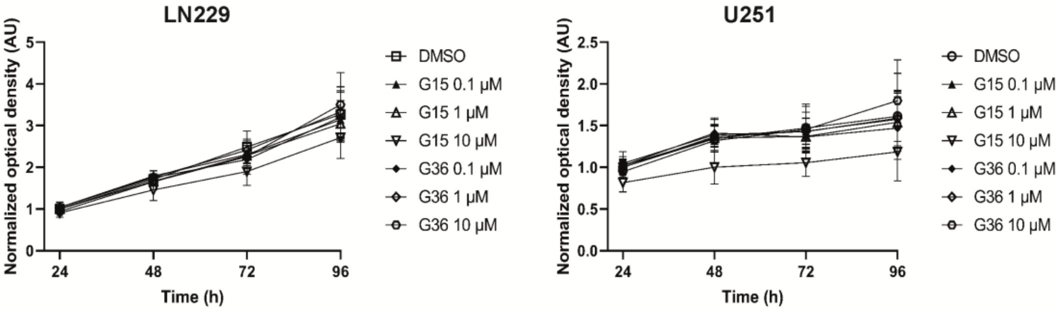

Supplementary Figure S1: G15 or G36 GPER antagonists had no effect on GBM cell proliferation. LN229 and U251 cells were treated with indicated concentrations of G15 or G36 for 96 h, and the cell number was determined using MTT assay. N=4. No difference was observed compared to DMSO-treated control cells. Data are presented as the mean ± SD. The level of significance was determined using Student's t-Test.

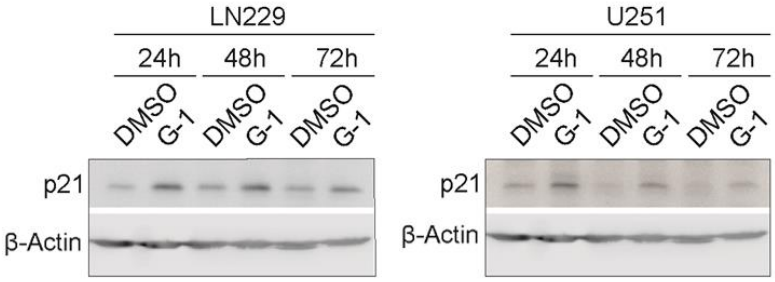

Supplementary Figure S2: G-1 increased p21 protein expression. Representative images of p21 expression assessed by western blotting from LN229 and U251 cells treated for 24 h, 48 h or 72 h with 1 μM G-1 or DMSO as control. β-actin was used as the reference protein.

Supplementary Video S1: G-1 altered GBM cell morphology as assessed by time-lapse video microscopy. U251 or LN229 cells stably expressing eGFP were allowed to attach for 24 h and then cultured for another 72 h in the presence of 1 μM G-1 or DMSO as vehicle. Imaging was performed every 2 h.
